# Effects of Living Cover on the Soil Microbial Communities and Ecosystem Functions of Hazelnut Orchards

**DOI:** 10.3389/fpls.2021.652493

**Published:** 2021-03-25

**Authors:** Wenxu Ma, Zhen Yang, Sihao Hou, Qinghua Ma, Lisong Liang, Guixi Wang, Chunli Liang, Tiantian Zhao

**Affiliations:** ^1^Key Laboratory of Tree Breeding and Cultivation of the State Forestry and Grassland Administration, Research Institute of Forestry, Chinese Academy of Forestry, Beijing, China; ^2^Hazelnut Engineering and Technical Research Center of the State Forestry and Grassland Administration, Beijing, China; ^3^National Hazelnut Industry Innovation Alliance, Beijing, China; ^4^Liaoning Agricultural Technical College, Yingkou, China

**Keywords:** living cover, *Vulpia myuros*, microbial communities, ecosystem functions, hazelnut orchard

## Abstract

Living cover is an important management measure for orchards in China, and has certain influences on soil properties, microorganisms, and the micro-ecological environment. However, there are few studies on the effects of living cover on the soil changes in hazelnut orchards. In this study, we compared the soils of living cover treatments with *Vulpia myuros* and the soils of no cover treatments, and analyzed the observed changes in soil properties, microorganisms, and microbial functions by using high-throughput ITS rDNA and 16S rRNA gene Illumina sequencing. The results demonstrated that the total organic carbon content in the 20–40 cm deep soils under the living cover treatments increased by 32.87 and 14.82% in May and July, respectively, compared with those under the no cover treatments. The living cover treatment with *V. myuros* also significantly increased the contents of total phosphorus (TP), total nitrogen (TN), available phosphorus (AP), and available potassium (AK) in the soil samples. Moreover, the influence of seasons was not as significant as that of soil depth. The living cover treatment also significantly improved the soil enzyme activity levels. The results demonstrated that Ascomycota, Mortierellomycota and Basidiomycota were the dominant fungal phyla in all samples, while Proteobacteria, Actinobacteria, Acidobacteria, Firmicutes, and Chloroflexi were the dominant bacterial phyla, but the different treatments impacted the compositions of fungal and bacterial communities. Principal component analysis (PCA) showed that living cover with *V. myuros* significantly changed the soil fungal community structures whereas the bacterial community structures may be more sensitive to seasonal changes. At the microbial functional level, the living cover treatment increased the fungal operational taxonomic units (OTUs) of symbiotrophs and decreased that of pathotrophs. According to this study, we believe that the application of a living cover with *V. myuros* has a favorable regulating influence on soil properties, microbial communities and microbial function. This treatment can also reduce the use of herbicides, reduce the cost of orchard management, and store more carbon underground to achieve sustainable intensification of production in hazelnut orchards, so it can be considered as a management measure for hazelnut orchards.

## Introduction

Hazelnut, a shrub or small tree belonging to *Corylus* L. in Betulaceae, is one of the four largest nuts in the world, together with the walnut, almond and cashew. Hazelnut trees have high ecological and economic value and are often used as a tree species for landscaping and soil and water conservation measures. *Corylus heterophylla* Fisch. × *Corylus avellane* L. is the main cultivated variety in China and has excellent characteristics, such as large fruit, high yield, strong adaptability, and high nutritional value; this variety, combines the advantages of *C. heterophylla* and *C. avellane* (Wang, 2018), and has been recognized by most producers in China (Luo et al., 2013; Zhu et al., 2014). In the process of planting hazelnut trees, clean tillage is usually carried out in orchards. Although this management practice can reduce nutrient competition between weeds and fruit trees, it also directly exposes the soil in orchards, which easily leads to surface runoff in the rainy season and reduces the soil nutrient supply capacity and causes soil hardening, soil erosion, organic matter loss, ecological environment damage, and other problems (Tebrügge et al., 1994; Lal, 1995; Shen, 2019).

The interplanting of grass in orchards is a kind of agroforestry system that has developed rapidly in recent years (Ishii et al., 2007; Triplett Jr and Dick, 2008). Previous studies tried to use *Trifolium repens* and *Lolium perenne* as interplanting grasses in orchards; these species can effectively inhibit soil and water loss. However, due to their growing periods being close to those of deciduous fruit trees, the interplanting of these species will inevitably lead to water and fertilizer competition (Qi et al., 2005; Liu et al., 2014; Rong et al., 2014; Wang et al., 2014). *V. myuros*, a perennial grass with a plant height of ~50 cm and dense growth, functions as conservation grass in deciduous orchards. This species germinates in September every year, falls naturally when it grows to ~50 cm and dies in June. During decomposition, the decomposing materials are mostly water-soluble substances that, can inhibit the growth of other weeds; thus, there is no need to cut the grass in summer, saving labor (Meyer et al., 1992; Cleland et al., 2006; Brown and Rice, 2010). After decomposition, the decomposed materials can also provide nutrients for fruit trees and improve the physical and chemical properties of the soil (Cleland et al., 2006; Krahulec and Nesvadbova, 2007). In addition, rattan grasses, which have fibrous root systems, can maintain soil moisture, prevent soil erosion, and stabilize the soil structure (Heeraman et al., 2001; Ishii et al., 2007; Wang et al., 2011). *V. myuros* and hazelnuts have different fertilization periods needed for their growth, which can meet the requirements for green fertilization in winter. Yang et al. found that July to October is the main period of *V. myuros* decomposition; this decomposition, can provide nutrients for the growth of fruit trees, instead of the grasses competing with fruit trees for nutrients (Yang et al., 2015). At present, *V. myuros* is mostly used in the planting of fruit trees and green fertilizers, and the application of *V. myuros* intercropping in hazelnut orchards has not been reported. Therefore, in this study, we used *V. myuros* as the living cover material.

In this study, we researched the soil properties (soil pH, soil bulk density, soil porosity, soil water content, nutrient contents, and enzyme activities), microbial diversity, community structure, and functional prediction at different depths (0–20 cm, 20–40 cm) under two different treatments (no cover, living cover) in spring and autumn. Soil microbial communities that displayed obvious differences between the two treatments were recognized by the linear discriminant analysis effect size tool (LEfSe, http://huttenhower.sph.harvard.edu/galaxy/root?tool_id=lefse_upload). The relationships between soil microbial community compositions and soil properties were discussed by Spearman correlation analysis. The objectives of this study were to (1) compare the differences in soil properties and microbial community compositions and functions between living cover treatment and no cover treatment and (2) study whether living cover treatment is beneficial to the development of soil micro-ecology. The results of this study can not only reveal the influence of living cover management with *V. myuros* on hazelnut orchards, but also provide theoretical support for the scientific management of hazelnut orchards.

## Materials and Methods

### Study Site and Soil Sampling

Our study was conducted in Yingkou (40°11′24″ N, 122°9′30″ E), Liaoning Province, China. This location has a continental temperate monsoon climate. The average annual rainfall is 700 mm, and the average annual temperature is 9.8°C. According to the (WRB, 2014), the orchard soil type was mainly clay loam (WRB, 2014). The main properties of the hazelnut orchard soil before the experiment were as follows: pH (5.90), total organic carbon (TOC, 11.37 g/kg), total nitrogen (TN, 0.62 g/kg), and total phosphorus (TP, 0.79 g/kg). The variety of hazelnut trees planted in this experiment was “Dawei” (*Corylus heterophylla* Fisch. × *Corylus avellane* L.), and the age of the trees was 3 years, and the row spacing × plant spacing was 4 × 3 m. There were two treatment methods used in this experiment: no cover (N) and living cover with *V. myuros* (L). Each treatment consisted of three randomly arranged plots, each of which was ~288 m^2^ (18.0 × 16.0 m). Samples were taken in May and July, and soil from the upper (A: 0–20 cm) and deeper (B: 20–40 cm) layers was collected each time. For the living cover treatments, 1.6-m-wide living covers were planted between rows. Clean cultivation was carried out under the trees. The covered crops were seeded in October 2016, with a sowing rate of 20 kg per ha of *V. myuros*. All treatments used the same fertilization method, which included 750 kg urea fertilizer and 1000 kg manure compost ha^−1^ · year^−1^. Soil samples were collected from the orchard on May 10th and July 25th in 2019 after the orchard was covered for 3 years. Six random soil samples were collected and mixed at depths of A and B between the rows in each plot. Finally, a total of 24 samples were obtained [two treatments (N: no cover treatment, L: living treatment) × two seasons (summer, autumn) × two depths (A: 0–20 cm, B: 20–40 cm) × three replicates]. The subsequent treatment of samples was consistent with the methods of previous studies (Qian et al., 2015; Zhang et al., 2020).

### Soil Physical and Chemical Properties

The soil pH was determined by the pH meter, and the ratio of water to soil was 2.5:1 (Qian et al., 2015). The soil water content, soil bulk density, and soil porosity were measured according to Soil Physical and Chemical Analysis (Institute of Soil Science, 1978). The K_2_CrO_4_ oxidation method was used to determine the TOC, and the Kjeldahl method was used to measure the TN. For the determination of the TP in the soil, the NaOH alkali fusion-atomic absorption method was adopted. The Olsen method was used to measure the soil AP. A flame photometer was used to determine the soil AK after NH_4_OAc extraction. The URE, CAT, ALP, and INV activities of the soil were determined according to the methods outlined in Qian et al. (2014). The DHA activity of the soil was determined by the soil enzyme kit from Solarbio Science and Technology Co. (Beijing, China) according to the methods of Kumar and Chaudhuri (2013).

### DNA Extraction and Polymerase Chain Reaction Amplification

The methods for extraction of microbial DNA from 24 soil samples, concentration and purification of the total DNA, and inspection of the DNA quality were described in Zhang's previous research (Zhang et al., 2020). The DNA samples were amplified in V5–V7 hypervariable regions in bacteria and 16S rRNA was amplified with primers 799 F (5′-AACMGGATTAGATACCCKG-3′) and 1193 R (5′-ACGTCATCCCCACCTTCC-3′), while the DNA samples amplified in the ITS-1 region in fungi were amplified with the primers IT1F (5′-CTTGGTCATTTAGAGGAAGTAA-3′) and IT2R (5′-GCTGCGTTCTTCATCGATGC-3′) by PCR (GeneAmp 9700, ABI, USA). The steps used in PCR have been described in detail in previous studies. The resulting PCR products were extracted from a 2% agarose gel and further purified using the AxyPrep DNA Gel Extraction Kit (Axygen Biosciences, Union City, CA, USA) and quantified using QuantiFluor™-ST (Promega, USA) according to the manufacturer's protocol (Li et al., 2018).

### Illumina MiSeq Sequencing and Data Processing

The sequencing of purified amplicons was described in the previous study (Zhang et al., 2020). Purified amplicons were pooled in equimolar amounts and paired-end sequenced (2 × 300) on an Illumina MiSeq platform (Illumina, San Diego, USA) according to the standard protocols by Majorbio BioPharm Technology Co. Ltd. (Shanghai, China). The original readings were uploaded to the NCBI Sequence Read Archive (SRA) database (Study accession number: SRP278043; BioProject ID: PRJNA657994). Trimmomatic was used to demultiplex and quality-filtere the raw fastq files, and FLASH was used to merge the files. The operational taxonomic units (OTUs) were clustered by UPARSE (version 7.1 http://drive5.com/uparse/), and the similarity cut-off value was 97%. The chimaeric sequences were identified and removed by UCHIME. According to the RDP Classifier algorithm, the classification of each ITS gene sequence was analyzed against the Unite database (version 7.2; http://unite.ut.ee/index.php) by using a confidence threshold of 70%. Then the taxonomy of each 16S rRNA gene sequence was analyzed by the RDP Classifier algorithm (http://rdp.cme.msu.edu/) cross referenced with the Silva (SSU123) 16S rRNA database (Li et al., 2018; Zhang et al., 2020).

### Statistical Analysis

One-way ANOVA of soil properties was carried out by SPSS (version 26.0; SPSS, Chicago, IL, USA). In all analyses, the significance was evaluated by Tukey's test (*p* < 0.05). The largest axis length was 3.38 at the OTUs level and 1.62 at the class level in the detrended correspondence analysis (DCA). Therefore, redundancy analysis (RDA) was carried out by using Monte Carlo permutations (permu = 999) to test the significance of the soil properties. According to the functions of envfit (permu = 999) and vif.cca, soil properties were selected, and the soil properties with *p* > 0.05 or vif > 10 were eliminated from the following analysis. The vif values of SWC, SP, TOC, URE, CAT, and ALP were higher than 10 and thus were eliminated. ANOSIM and RDA were carried out in R for statistical calculations (Core Team, 2015). The Canoco program for Windows 4.5 (Biometris, Wageningen, The Netherlands) was used for principal component analysis (PCA). Using FUNGuild (http://www.stbates.org/guilds/app.php) which is used as a tool to classify and analyse fungal communities through microecological guides based on the published literature or authoritative website data, the fungi were divided into pathotrophs, symbiotrophs and saprotrophs (Nguyen et al., 2016). The PICRUSt (http://huttenhower.sph.harvard.edu/galaxy/root?tool_id=PICRUSt_normalize) pipeline was used on the Galaxy server to predict the functional potential of bacteria (Langille et al., 2013).

## Results

### Soil Properties and Nutrient Contents

As shown in Supplementary Tables 1 and 2, the water content and pH of the soil in the living cover treatments were considerably (p < 0.05) higher than those of in the no cover treatment. The water contents of the upper layer of the soil in the living cover treatment soil were 11.59 and 19.43% higher than those in the no cover treatment soil in May and July, respectively. In the deeper soil, the water contents were 28.94 and 48.05% higher in the living cover treatment soil than in the soil with no cover treatment in May and July, respectively. In May, there was no difference in bulk density between the two treatments. The living cover treatment soil had a significantly lower bulk density than the no cover treatment soil in July. The soil porosity values had similar performances. In May, the pH values of the upper and deeper soil layers under the living cover treatment were 14.53 and 9.88% higher than those under the no cover treatment, respectively. In addition, in July, the pH values were 6.37 and 10.70% higher in the upper and deeper soil levels, respectively, under the living cover treatment than under the no cover treatment. The soil TOC content under the living cover treatment was higher than before (11.37 g/kg), but no significant (*p* > 0.05) effect was observed under the no cover treatment. Compared with that under the no cover treatment, the TOC content of the upper soil under the living cover treatment in the upper soil increased by 18.02 and 17.21% in May and July, respectively, while that of the deeper soil increased by 32.87 and 14.82%, respectively. In May, the levels of TP, TN, AP, and AK increased by 13.20, 8.89, 13.84, and 13.79%, respectively, under the living cover treatment compared to those under the no cover treatment, while in July, the values were 12.17, 12.12, 16.82, and 23.10% higher under the living cover treatment than under the no cover treatment. With the change in season, the TN content decreased in the two soil layers, but the TP, AP, and AK contents increased. In May, the C/N ratio of the deeper soil in the living cover treatment was significantly higher than that in the no cover treatment. Although the other factors displayed no significant differences between the no cover treatment and the living cover treatment, the values in the living cover treatment were slightly higher than those in the no cover treatment.

### Soil Enzymes

The living cover treatment significantly (*p* < 0.05) improved the soil urease (URE), catalase (CAT), alkaline phosphatase (ALP), invertase (INV), and dehydrogenase activity contents (Supplementary Table 3). In the upper soil, the soil urease activity under the living cover treatment (11.60 ± 0.13 IU/g) was 22.88% greater than that under the no cover treatment (9.44 ± 0.07 IU/g) in May and 49.15% greater in July. The soil catalase activity under the living cover treatment was 12.28% higher than that under the no cover treatment in May and 33.38% higher in July. The soil alkaline phosphatase activity under the living cover treatment was 5.33% higher than that under the no cover treatment in May and 11.76% higher in July. The soil invertase activity under the living cover treatment was 50.94% higher than that under the no cover treatment in May and 21.43% higher in July. In the deeper soil, the soil urease activity under the living cover treatment was 1.05 times that under the no cover treatment in May and 1.10 times that in July. The soil catalase activity under the living cover treatment was 1.30 times that under the no cover treatment in May and 1.11 times that in July. The soil alkaline phosphatase activity under the living cover treatment was 1.22 times that under the no cover treatment in May and 1.17 times that in July. The soil invertase activity of the living cover treatment was 1.52 times that of the no cover treatment in May and 1.13 times in July. The enzyme activities of each treatment were higher in the upper soil than in the deeper soil. The levels of soil dehydrogenase activity under the living cover treatment were 2.63 times those under the no cover treatment in the 0–20 cm soil layer and 2.92 times in the 20–40 cm soil layer in July.

### Fungal Community Diversity

The Shannon index (H'), richness index (S), and evenness index (E') of the fungal communities were evaluated with Illumina MiSeq high-throughput sequencing data (Supplementary Table 4). Most living cover treatments remarkably increased the levels of H', S, and E' in the soils. The living cover treatment significantly (*p* < 0.05) increased the richness index (S). The richness index of the orchards in the living cover treatment was 38.91% higher than that in the no cover treatment in spring and 38.80% higher in summer. Among the no cover treatments and living cover treatments, there was no remarkable (*p* > 0.05) difference in the three diversity indexes between the upper and deeper soil layers. The season had no obvious influence on the three indexes. The S level of the living cover treatment was the highest in the upper soil in May (189.7 ± 6.4); this value was considerably (*p* < 0.05) higher than those obtained under the no cover treatments.

### Bacterial Community Diversity

The Shannon index (H'), richness index (S), and evenness index (E') of the bacterial community were evaluated by the Illumina MiSeq high-throughput sequencing data (Supplementary Table 5). All living cover treatments improved the H', S, and E' in the soils, but there was no obvious (*p* > 0.05) difference in these three diversity indexes between the no cover treatments and living cover treatments except NB_May (392.7 ± 24.8) and LB_May (440.7 ± 33.8). The season and the soil depth had no significant (*p* > 0.05) influences on the three indexes. The S level under the living cover treatment (440.7 ± 33.8) was the highest in the deeper soil in May.

### Fungal Community Structure

**Figure 1:**
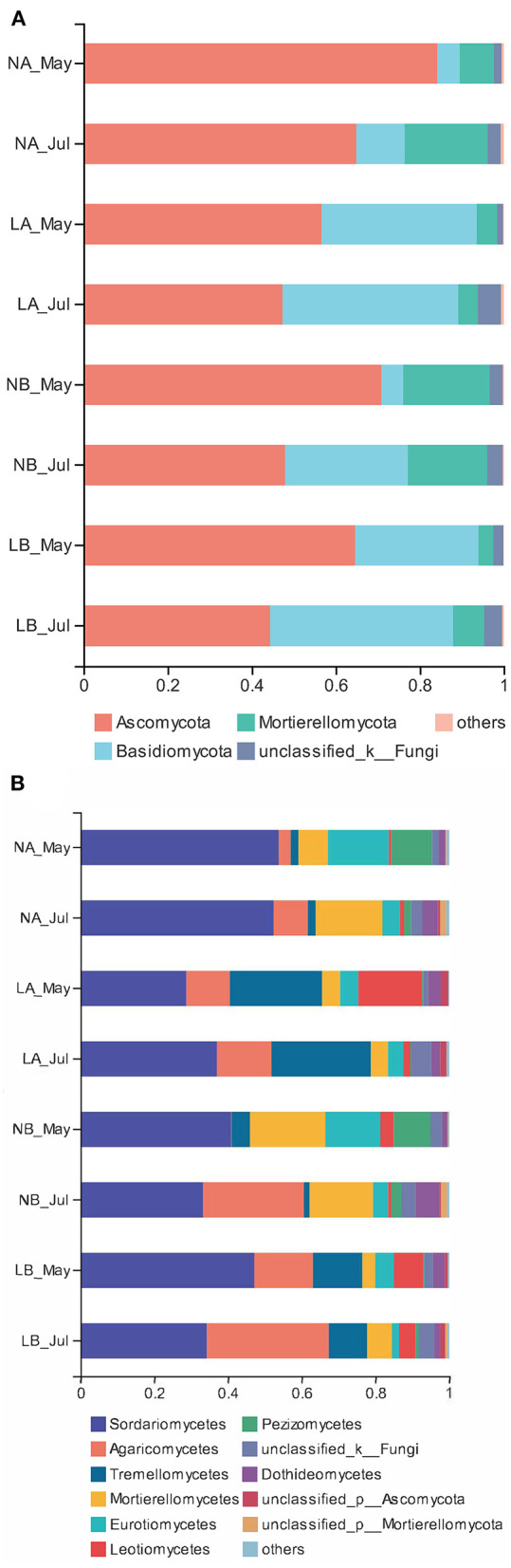
Relative abundances of fungal phyla and classes in the no cover treatments and living cover treatments. **(A)** The structure of the fungal community at the phylum level. **(B)** The structure of the fungal community at the class level.

**Figure 2:**
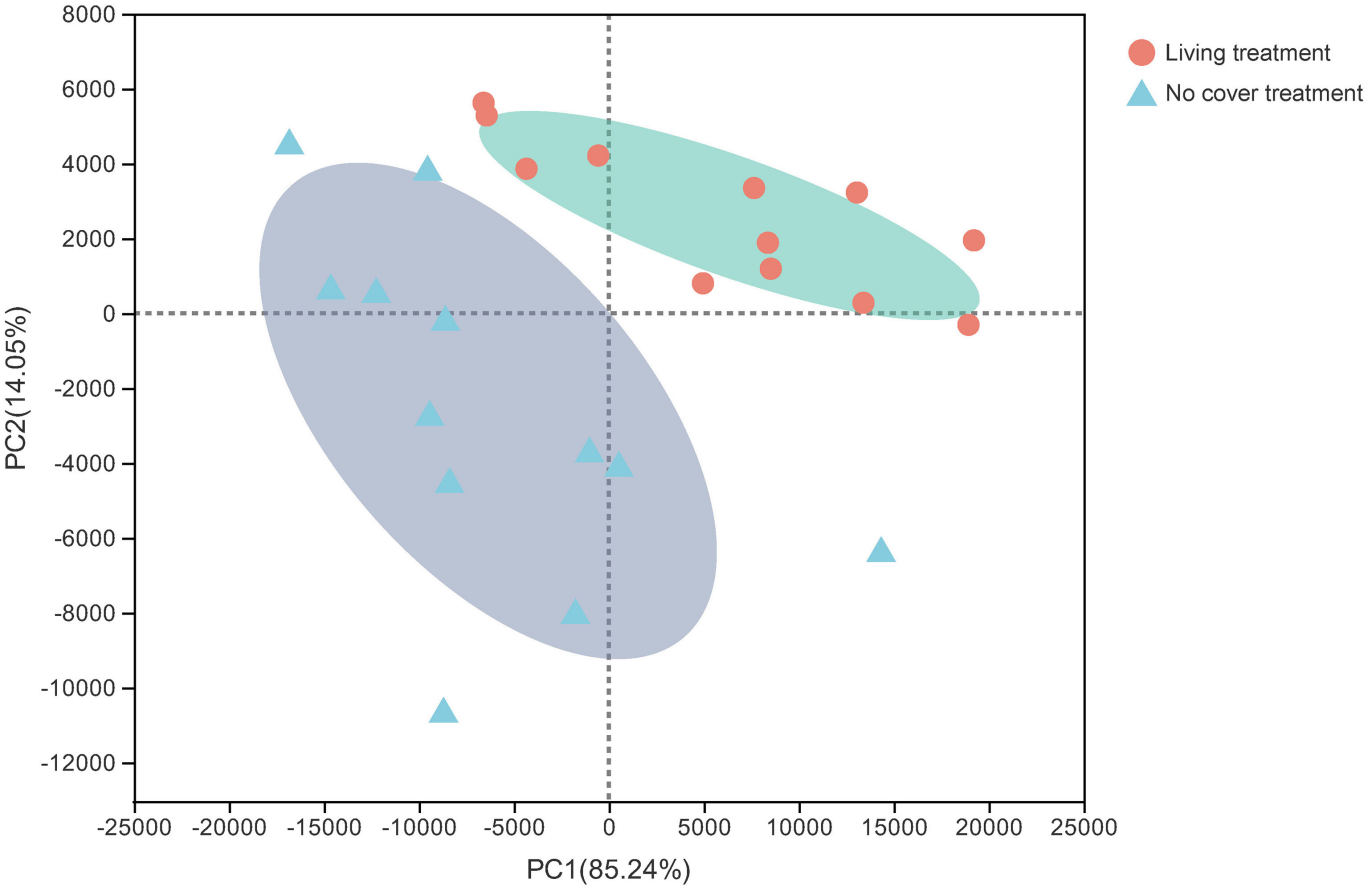
PCA of fungal communities. The values on axes 1 and 2 are the interpretable percentages of the corresponding principal components.

To standardize the different sequencing depths, each sample was randomly selected from 44,412 reads to analysis (Supplementary Table 6). Ascomycota (60.16%) and Basidiomycota (25.36%) were the main fungal communities (Figure 1). Unclassifled_k_Fungi was represented 3.14% of all sequences. The relative abundances of Ascomycota and Mortierellomycota under the living cover treatments decreased compared with those under the no cover treatments, but the relative abundances of Basidiomycota increased. PCA also showed the difference between the no cover and living cover treatments, and the soil samples in the different treatments were obviously separated. Two principal components were determined, which clarified 99.29% of the total variance in the dataset (Figure 2). Sequences from Ascomycota dominated in the upper soil samples (63.31%) and deeper soil samples (57.01%) collected under both treatments (no cover and living cover) (Figure 1A). With the change in season, the relative abundance of Ascomycota decreased, whereas that of Basidiomycota increased under the two treatments (no cover and living cover) at the phylum level, but there was no obvious change in Mortierellomycota (Figure 1A). The relative abundance of Ascomycota in spring was higher than that in summer, but the relative abundance of Basidiomycota was the opposite.

The overall fungal community was dominated by Sordariomycetes (40.92%) and Agaricomycetes (14.43%) (Figure 1B) at the class level. In both treatments (no cover and living cover), the classes Agaricomycetes and Tremellomycetes represented 18.89 and 18.95% of the sequences in the samples collected from the living cover treatments, respectively, whereas these classes represented only 9.98 and 2.70% of the sequences in the samples collected from the no cover treatments (Figure 1B). The classes Mortierellomycetes and Eurotiomycetes were represented by 15.93 and 10.07% of the sequences in the no cover treatment, whereas these classes represented only 4.96 and 4.02% of the sequences in the living cover treatment (Figure 1B). With seasonal changes, the relative abundances of Sordariomycetes in the living cover treatments decreased, while those in deeper soil increased. The relative abundances of Agaricomycetes, Tremellomycetes and Leotiomycetes under the living cover treatments increased compared with those under the no cover treatments in both the upper soil and deeper soil, while the situations for Mortierellomycetes, Eurotiomycetes and Pezizomycetes were the opposite (Figure 1B).

### Bacterial Community Structure

**Figure 3:**
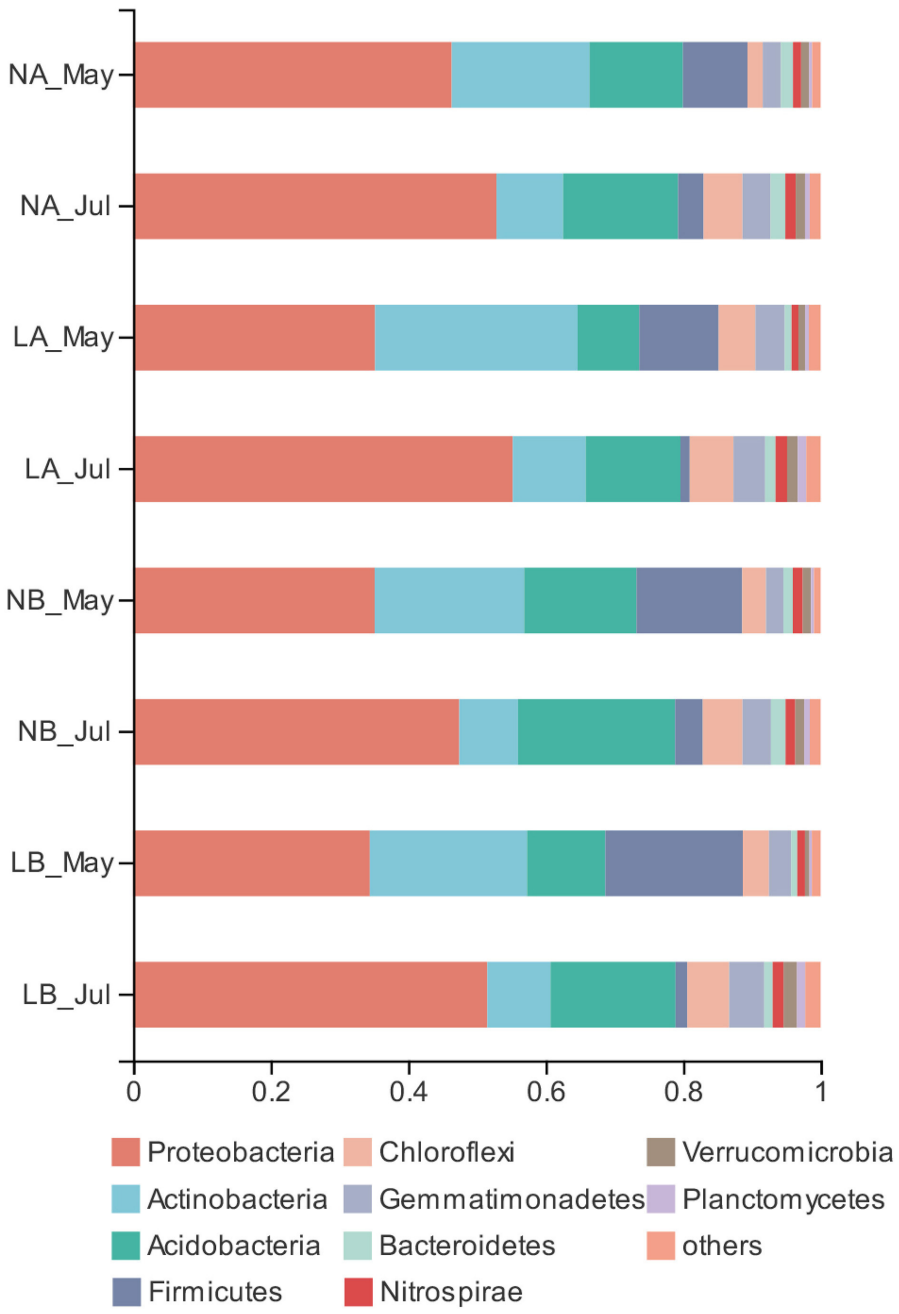
Relative abundances of bacterial phyla in the no cover treatments and living cover treatments.

To standardize the different sequencing depths, each sample was randomly selected from 14,275 reads to analysis (Supplementary Table 7). The dominant bacteria were Proteobacteria (44.78%), Actinobacteria (16.48%), and Acidobacteria (15.22%) among all samples (Figure 3). Living cover did not show a considerable effect on the relative abundances of the phyla except Elusimicrobia (Supplementary Figure 1). The relative abundances of Acidobacteria under the living cover treatments decreased compared with those under the no cover treatments, while the relative abundances of Actinobacteria increased, but neither of these two phyla demonstrated a significant correlation. There was no significant (*p* > 0.05) difference between the deeper soil and upper soil layers, but there were significant differences between seasons at the phylum and class levels (Supplementary Figures 2, 3).

**Figure 4:**
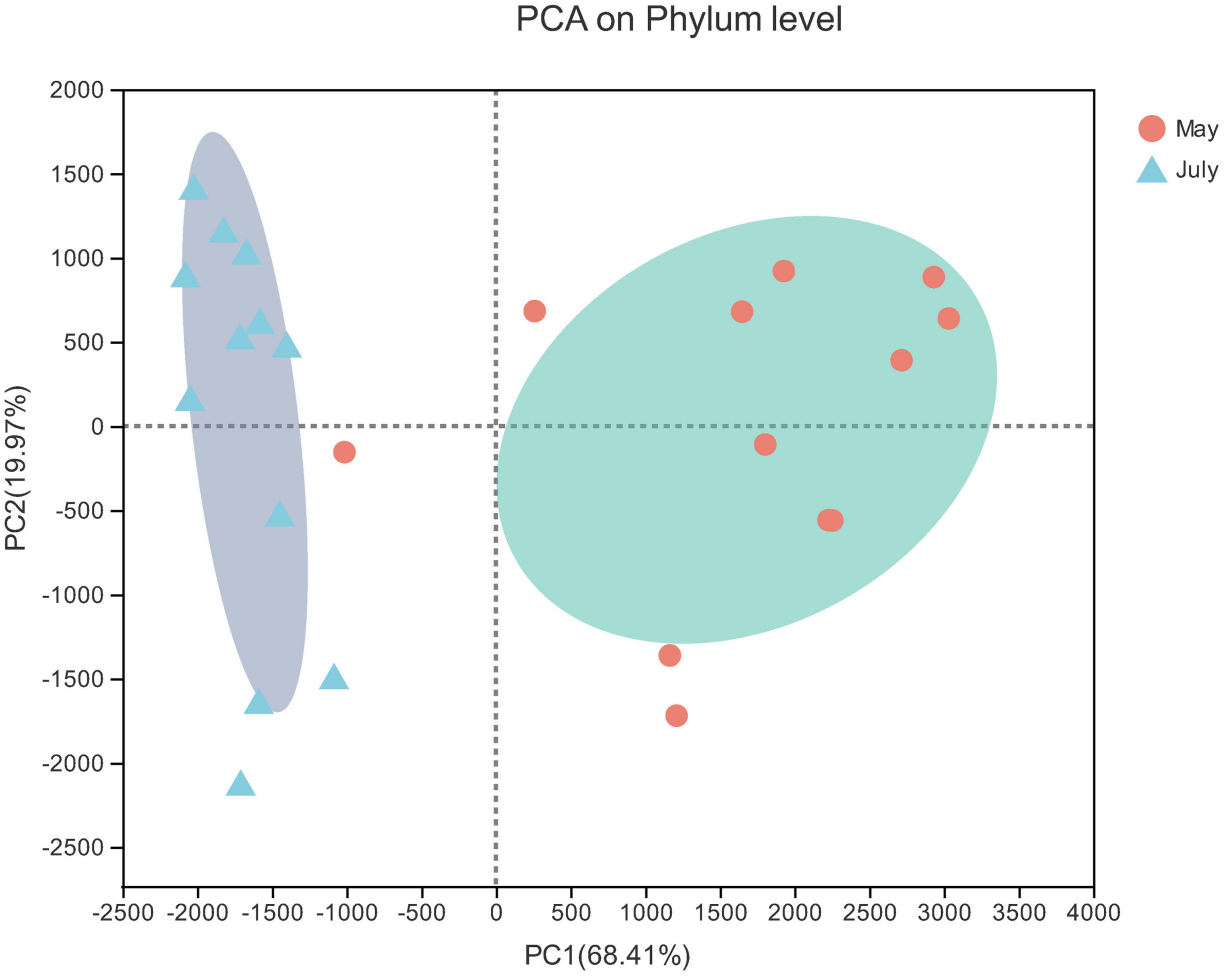
PCA of bacterial communities. The values on axes 1 and 2 are the interpretable percentages of the corresponding principal components.

The PCA results showed obvious differences between the soil samples collected in spring and summer. Two principal components were determined that explained 88.38% of the total variance in the dataset (Figure 4). Sequences from Proteobacteria dominated in spring soil samples (37.79%) and summer soil samples (51.77%) (Figure 3). The relative abundances of Proteobacteria and Acidobacteria were highest in summer and lowest in spring, while Actinobacteria and Firmicutes reached their highest abundance in spring.

### Relationship Between Microbial Community Structures and Soil Properties

**Figure 5:**
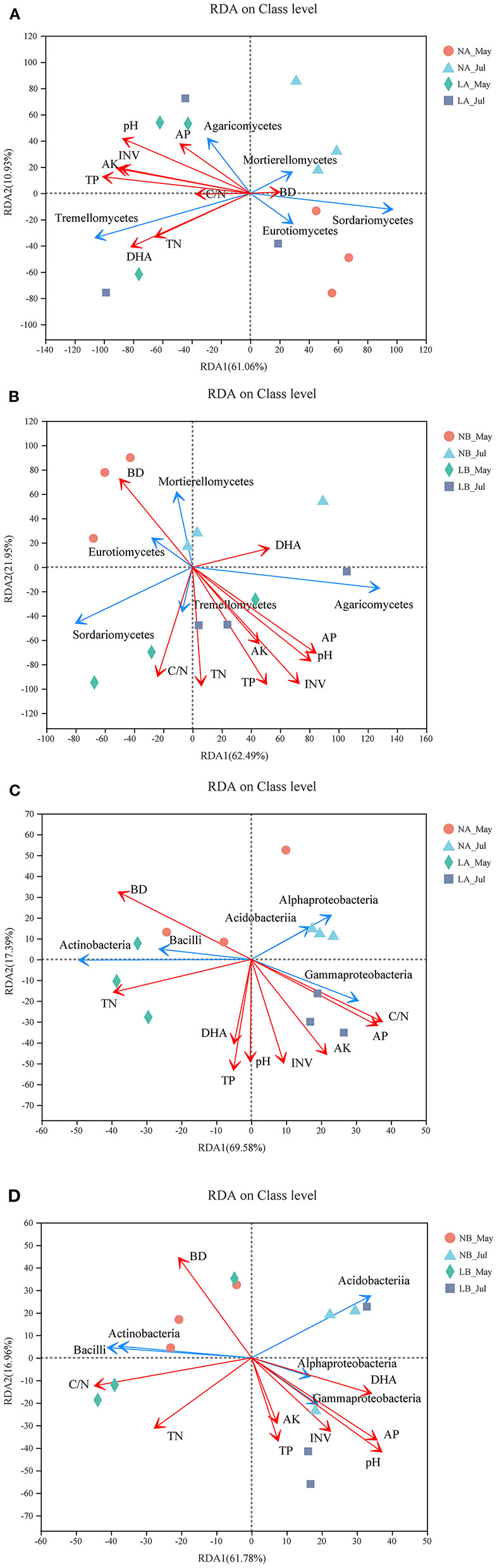
Redundancy analysis (RDA) of MiSeq data and soil properties. **(A)** RDA of fungal communities in the 0–20 cm layer. **(B)** RDA of fungal communities in the 20–40 cm layer. **(C)** RDA of bacterial communities in 0–20 cm layer. **(D)** RDA of bacterial communities in 20–40 cm layer. The red lines with arrows indicate soil properties, and the blue lines with arrows indicate the top five fungi or bacteria classes. The values on axes 1 and 2 are the interpretable percentages of the corresponding principal components.

The presence of a cover treatment changed the observed microbial community structures and soil properties. After removal of the redundant variables, nine soil properties were chosen for RDA. As shown in Figures 5A,B, pH, TP, C/N, AK, INV, and DHA significantly affected the fungal community structures in the upper soil layers, and all soil properties except AK and DHA obviously (*p* < 0.05) impacted the fungal community compositions in the deeper soil layers. All soil properties except DHA remarkably (*p* < 0.05) impacted the bacterial community compositions in the upper soil layers, while pH and AP dramatically (*p* < 0.05) impacted the bacterial community compositions in the deeper soil layers (the relevant *p*-value can be seen in Supplementary Table 8). Regarding the angles between the arrows connecting the lines representing different soil properties, those of TP, pH, INV, AK, and AP were always small, which showed that they had good correlations and were positively correlated in each treatment.

### Prediction of the Community Functions of Soil Fungi and Bacteria

**Figure 6:**
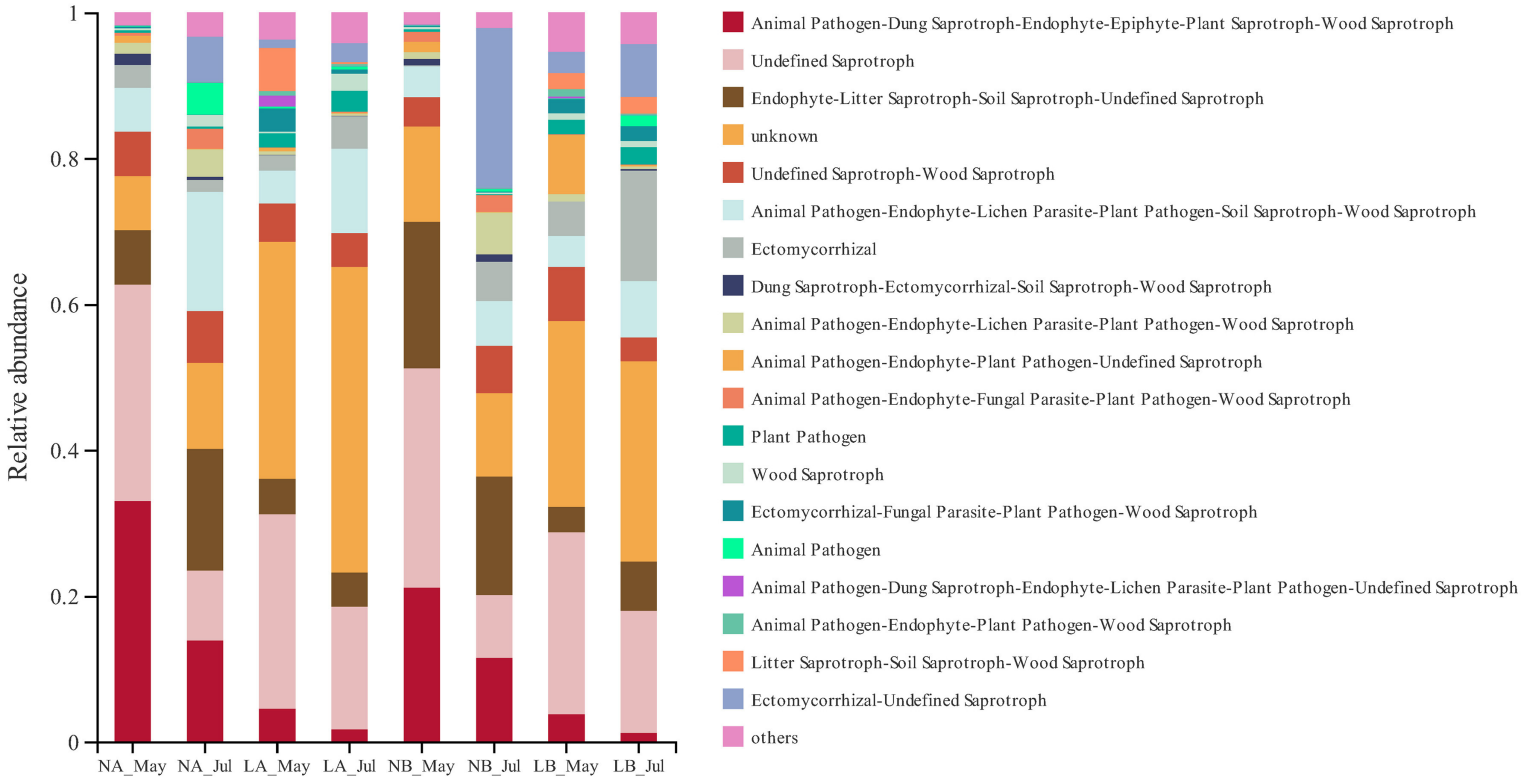
Functional features of fungal communities, as inferred by FUNGuild.

**Figure 7:**
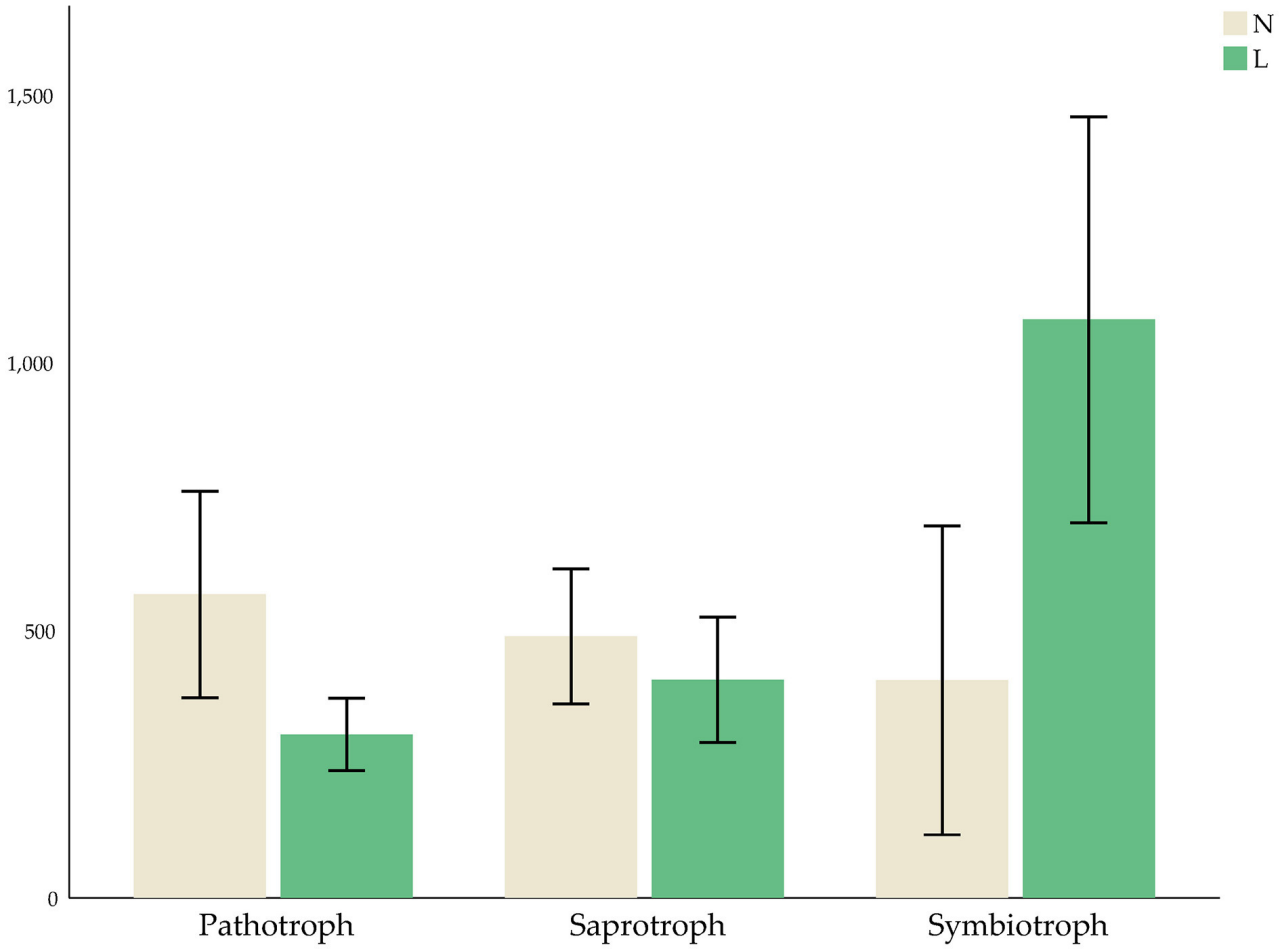
Average OTUs values of three kinds of fungal nutritional modes.

The micro-ecological functions of fungi and bacteria in the soil of hazelnut orchards under the no cover and living cover treatments were studied by analyzing the fungal and bacterial communities with FUNGuild and PICRUSt1. The guilds identified in the present study are listed in Figure 6, and the results demonstrated that the functional prediction results for fungi were related to different treatments. Three nutritional patterns (pathotrophs, saprotrophs, and symbiotrophs) accounted for ~36.69, 49.80, and 2.59% of the no cover treatment fungal OTUs, respectively, whereas under the living cover treatments, these patterns accounted for 19.76, 41.51, and 6.89%, respectively (Figure 7). There was no obvious divergence between the no cover treatments and living cover treatments according to the analysis of the bacterial communities in the Cluster of Orthologous Groups (COG) database by PICRUSt1 (Supplementary Figure 4).

## Discussion

One of the main considerations in the technology of living cover treatments involves the water distribution between the living cover and fruit trees. Some living covers compete with fruit trees for water, which adversely affects the growth of fruit trees (Unger, 1998; Zhao and Li, 2006; Wang et al., 2009). In the present study, because of the relatively abundant rainfall that occurred before the two sampling periods, the competition for water between the living cover with *V. myuros* and the hazelnut trees was very weak. Additionally, as shown in Supplementary Tables 1 and 2, living cover with *V. myuros* may also be beneficial to water collection and storage. The soil bulk density index is a quantitative criterion used to measure whether a given soil tightness is suitable for crop root growth. A low soil bulk density indicates that the soil is loose with good permeability and high fertility. This characteristic can also be reflected by the SP index and the content of organic matter. The fibrous roots of *V. myuros* may be one of the reasons for the observed decrease in soil bulk density, and the decomposition of litter in soil may also cause the decrease in soil bulk density.

Studies on apples and teas showed that pH decreased with increasing planting years in orchards (Ding and Zeng, 2014; Su et al., 2014). Under acidic conditions, the ability of plant roots to absorb water and nutrients is limited, and the growth and development of plants are inhibited. The accumulation of H^+^ in soil accelerates soil acidification, which destroys the plasma membrane of root cells and leads to nutrient loss (Yi et al., 2006). It can be clearly seen from Figure 1 that the observed pH values in the upper layer of the uncovered soil were lower after 3 years of planting than before planting, but the pH values were significantly increased after *V. myuros* was planted; the living environment of this type of living cover is able to meet the pH requirements (5.5–8.0) in the technical regulations for cultivation of Ping'ou hybrid hazelnuts (Research Institute of Forestry Chinese Academy of Forestry, 2013). In the no cover treatments, the leaching of cations may lead to a decrease in base saturations and increased exchange acidities; in contrast, decreases in soil pH and increased base saturations may be observed in the cover treatments. The use of nutrient ions by plentiful microbial communities in the rhizosphere of the root of living covers may also contribute to this trend (Haynes and Goh, 1980).

Plant litter covers the bases of trees or turns into soil, thus providing nutrient resources for soil through microbial decomposition (Marsh et al., 1996; Sanchez et al., 2003; Stork and Jerie, 2003). Annual fertilization is also an important way to improve soil nutrient concentrations. Studies have proven that living cover can improve soil nutrient levels by improving microbial activities in soils (Kamh et al., 1999; Stork and Jerie, 2003; Yao et al., 2005). The total nutrient level of a soil reflects the storage of soil nutrients, while the available nutrient level of a soil reflects the dynamic balance between plant absorption and soil mineralization (Wang et al., 2016). The reason the C/N under living cover treatments was higher than that under no cover treatments may be that the living cover treatments provided more litter and increased the organic matter contents of the soil. The increase in C/N also slows down the mineralization and decomposition of organic matter, which is beneficial to the accumulation of organic matter and improves the carbon sequestration capacity of soil. In this study, all the living cover treatments remarkably enhanced the TOC, TP and TN contents of the soil samples. All the living cover treatments obviously enhanced the AP and AK contents in the soils, revealing the positive effect of living cover plants on soil P and K. The reason for this result may be related to the composition of the microbial community, and Proteobacteria could be capable of exploiting labile carbon sources (Fierer et al., 2007; Kuramae et al., 2015). Actinobacteria contribute to the decomposition of organic matter and have the ability to break down complex substrates (Kamau et al., 2008; Nemergut et al., 2011). Mortierellomycota have the ability to solubilize phosphorus (Fröhlich-Nowoisky et al., 2015; Grzdziel and Gazka, 2019). Basidiomycota play an important role in degrading lignin under anaerobic conditions (Ivancevic and Karadelev, 2013). However, most of the above fungal or bacterial contents were higher under the cover treatments than under the no cover treatments. In summary, *V. myuros* had no competitive effects on hazelnut trees.

The soil urease activity, soil alkaline phosphatase activity and soil invertase activity exhibited a very good correlations with the TOC and TN soil contents. Enzyme activity in soil can be regarded as an important indicator of soil fertility, and plays a crucial role in maintaining and improving soil fertility (Zantua et al., 1977; Dick et al., 1988; Li, 1989; Yang and Wang, 2004; Lin et al., 2010; Burns et al., 2013). The results contrast with Xi et al.'s results (Xi et al., 2011; Qian et al., 2015), in which living covers with Leguminosae (*Coronilla varia* and *Medicago sativa*) in vineyards and apple orchards obviously increased the soil INV, URE, and ALP activities, whereas living covers with Gramineae had no substantial effects on the activities of these three enzymes. In this study, the results showed that the positive effects of living cover treatments on soil enzyme activities were better than those observed in the no cover treatments. The different soil enzyme activity expression levels may be related to the influences of living cover treatments on the soil microbial community compositions (Yao et al., 2005; Breulmann et al., 2012). The results obtained in this study were similar to those of Xu et al., who found that living covers with Gramineae had obvious influences on soil enzyme activities compared with no cover treatment (Xu et al., 2018). The possible reason for the improvement in the soil enzyme activity level was that litter and root exudates provided abundant nutrients for orchard soil microorganisms under the living cover treatments and changed the physical and chemical properties of the soil, thus improving the activities of microorganisms (Wardle et al., 2001; Hoagland et al., 2008; Smith, 2010).

As shown in Supplementary Table 3, the enzyme activities observed in the samples from the upper soil layers were remarkably higher than those in the samples from the lower soil layers under all treatments. Many research results have shown that soil enzyme activity decreases with increasing soil layer thickness. There may be two reasons for this result: on the one hand, the contents of soil organic matter and other nutrients that have great influences on enzyme activity decrease with increasing of soil profile depth; on the other hand, fine roots are mainly distributed in the surface soil, while *V. myuros* has fibrous root characteristics, and its roots are mainly concentrated in the upper soil. Therefore, the root concentration gradually decreased from top to bottom in the soil, so soil enzyme activity also showed a decreasing trend (Zhang and Yu, 1989; Zhang and Chen, 1997; Wang, 2006). In the process of this study, the growth of other grasses was not found in the living cover treatment with *V. myuros*. Therefore, *V. myuros* has positive effects on soil moisture retention, weed inhibition, soil nutrient accumulation, and soil enzyme activity promotion.

In this study, ITS and 16S rRNA Illumina MiSeq high-throughput sequencing data were used to measure the changes in fungal and bacterial communities in soil. Illumina MiSeq high-throughput sequencing data can reflect the genetic diversity of fungal and bacterial communities, and the Illumina MiSeq high-throughput sequencing analysis included the whole microbial community. As shown in Supplementary Table 4, living cover treatments obviously increased the diversity of fungal communities and functional structures in the soil samples. The PCA results also showed a clear separation between the soil samples collected under the different treatments (no cover and living cover). Living cover treatments can significantly improve the abundance and diversity of soil microorganisms. This result was consistent with previous research results (Mitchell et al., 2017; Zhang et al., 2020). However, there was no significant correlation between soil depth and seasonal changes. This result may be because the abundance and diversity of fungi were insensitive to the short changes in environmental factors caused by soil depth and seasonal changes, which may be the reason why many studies analyzed the changes in only the abundance and diversity of bacteria or nematodes caused by environmental changes (McCaig et al., 1999; Hoagland et al., 2008; Qian et al., 2015). As shown in Supplementary Table 5, the bacterial community diversity did not significantly differ between the two treatments, but it can be clearly known that the bacterial diversity of each soil layer in the living cover treatments was higher than that in the no cover treatments, and the bacterial diversity in the upper soil was higher than that in the deeper soil in both treatments. However, the rate of increase in the living cover treatments was slightly higher than that in the no cover treatments, which may be related to the fact that *V. myuros* has fibrous root characteristics mainly distributed in 0–20 cm soil layer.

Fungi are the main microbial group that decompose forest soil organic matter by producing enzymes (Baldrian, 2008). Our results demonstrated that Sordariomycetes and Mortierellomycetes may have important contributions to soil organic matter decomposition in the no cover treatments, while Agaricomycetes, Tremellomycetes, and Leotiomycetes might have been more important under the living cover treatments (Figure 1) (Schneider et al., 2012; Zhang et al., 2017). According to reported studies, some members of Agaricomycetes are related to most ectomycorrhizae, and it has been widely concluded that ectomycorrhizae can promote the growth of trees and the carbon cycle and are very important to temperate forests. Other class members are critical decomposers that can effectively decompose wood polymers (Chen et al., 2006; Ren and Mallik, 2006; Song et al., 2007; Aislabie et al., 2013; Kersten and Cullen, 2013). The most abundant genera of Agaricomycetes in the soils under the living cover treatments were *Tomentella, Paxillus, Inocybe*, and *Hymenogaster*, which are known as ectomycorrhizal fungi in the present study (Matheny, 2009; Tedersoo et al., 2010a). Lilleskov et al. suggested that *Tomentella* was related to nitrogen deposition and was an important part of the community structure under conditions of high overall nutrient availabilities (Lilleskov et al., 2002). Studies have shown that *Paxillus involutus* can degrade organic matter such as plant litter by overexpressing a number of oxidase transcripts (Rineau et al., 2012). Polyphenol oxidase and protease activities in plant litter inoculated with *P. involutus* were significantly improved and were 2–3 times higher than those in litter without a *P. involutus* treatment (Bending and Read, 1995). *Inocybe* and *Hymenogaster* also play very important roles in the growth and ecology of trees by forming ectomycorrhizal associations (Burgess et al., 1993; Tam, 1995; Akiyoshi and Keizo, 2001; Smit et al., 2001; Obase et al., 2006; Fierer et al., 2007; Tedersoo et al., 2010b; Kennedy et al., 2011; Ma, 2012). According to the functions of ectomycorrhizal fungi clarified in the above research, it was speculated that increases in fungal contents in living covers may lead to changes in soil nutrient enzyme activity levels. In the upper soil layers, the extracellular enzyme activities reached their peaks in summer when fresh grass litter accumulated. The RDA results (Figure 5) also showed that there was a favorable correlation between extracellular enzyme activity and the relative abundance of Tremellomycetes. The measured change in fungal community composition under living cover treatments may be an important factor leading to increases in the amount of organic matter available for recycling. Oligotrophia is a characteristic of members of Acidobacteria (Fierer et al., 2007), which explains why members of Acidobacteria can live in low-carbon and low-pH environments. This may demonstrate why the proportions of Acidobacteria were enhanced in the no cover treatments because no cover treatments had lower soil carbon and pH levels than the living cover treatments. This may also may demonstrate the influence of sampling depth, as deeper soil had lower soil carbon and pH levels than upper soil. Moreover, the ratio of Proteobacteria to Acidobacteria can be used to represent the nutritional status of the ecosystem, and if this ratio is low, *a critical soil nutritional status* is indicated (Smit et al., 2001). In the current study, this ratio was low in the no cover treatments, which showed that the no cover treatments could be regarded as having malnutrition statuses compared with the living cover treatments. This finding was consistent with the results of the RDA of environmental factors shown in Figures 5C,D. Seasonal changes had a significant impact on the community structures of bacteria, possibly because the sampling site belongs to a typical monsoon climate at medium latitudes, and the bacterial structures were highly sensitive to alternating seasonal periods (Lan et al., 2018).

Previous studies have shown that reducing farming can protect some fungal plant pathogens from high temperatures, limited water resources and interference (Bockus and Shroyer, 1998; Schroeder and Paulitz, 2006). Some studies have also shown that reducing cultivation can limit the movements of plant pathogen spores, maintain more microbial communities, and inhibit the invasion and establishment of plant pathogens (Bockus and Shroyer, 1998; Larkin, 2015; van Bruggen and Finckh, 2016). Interestingly, the results of FUNGuild showed the presence of living cover treatments decreased the number of pathotrophs and increased the number of symbiotrophs in this study, which was consistent with the previous research results.

Proteobacteria and Actinobacteria are copiotrophs that are characterized by preferential consumption of soil organic carbon pools and high nutritional demands (Fierer et al., 2007; Aislabie et al., 2013; Zhang et al., 2017). It is possible that there was little difference in the total amount of these two bacteria under each treatment, which led to similar prediction functions in each treatment. Studies in soil microcosms have shown that ectomycorrhizal fungal mycelia can reduce the activity of saprophytic bacteria (Olsson et al., 2010). However, the function of bacteria did not change significantly in this study, which may be related to the low OTUs contents of ectomycorrhizal fungi.

## Conclusion

In our study, we researched the influences of living cover, soil depth and seasonal changes on microbial diversity, community composition, and ecological functions in hazelnut orchard soil. The microbial compositions and ecological functions were affected by living cover treatments with *V. myuros* in hazelnut orchard management practices. The results showed that living cover treatments with *V. myuros* improved the physical and chemical conditions of soils in hazelnut orchards and caused great changes to the soil microbial diversity, community composition and ecological functions, especially in the fungal community, which reduced the OTUs of pathotrophs and increased that of symbiotrophs. The living cover treatments with *V. myuros* in hazelnut orchards could have more beneficial and diverse microecological environments than the no cover treatments. Therefore, the application of living covers with *V. myuros* could be a good management practice for the hazelnut orchards compared with no cover management, which can realize sustainable intensification of production in hazelnut orchards and provide certain theoretical support for the scientific management of hazelnut orchards. Furthermore, because the hazelnut tree did not reach a stable fruiting period during the study period, it is necessary to study the influence of living cover on the seed setting and other characteristics of the hazelnut tree in the future. Moreover, studying the reasons why the living cover treatments had no obvious effects on the differences in bacterial function will be indispensable. It is also necessary to study the effects of soil microorganisms under different cover treatments to identify the most suitable cover plants for hazelnut orchards in the future.

## Data Availability Statement

The datasets presented in this study can be found in online repositories. The names of the repository/repositories and accession number(s) can be found at: https://www.ncbi.nlm.nih.gov/sra/?term=SRP278043.

## Author Contributions

WM carried out the experiments, collected and organized data, and wrote manuscript. ZY and SH participated in the data analysis. QM and LL reviewed the manuscript and gave constructive suggestions. GW participated in the design experiment and guided the research. CL helped to do the experiment. The corresponding author TZ, put forward the basic hypothesis of this work, designed experiments, and helped organize the structure of manuscript. All authors read and approved the final manuscript.

## Conflict of Interest

The authors declare that the research was conducted in the absence of any commercial or financial relationships that could be construed as a potential conflict of interest.

## Supplementary Material

Supplementary Figure 1 The relative abundance of main bacterial community in soils under different treatments.

Supplementary Figure 2 The relative abundance of main bacterial community in phylum level under different treatments.

Supplementary Figure 3 The relative abundance of main bacterial community in class level under different treatments.

Supplementary Figure 4 Bacterial community functional features inferred by PICRUSt1.

Supplementary Table 6 Fungi OTUs.

Supplementary Table 7 Bacteria OTUs.

Supplementary Table 8 Envfit environmental characteristics.
