# Supplementary figures and images for: Effects of Living Cover on the Soil Microbial Communities and Ecosystem Functions of Hazelnut Orchards
Source: Front Plant Sci. 2021 Mar 25;12:652493. doi: 10.3389/fpls.2021.652493 (PMC8033216; doi:10.3389/fpls.2021.652493)

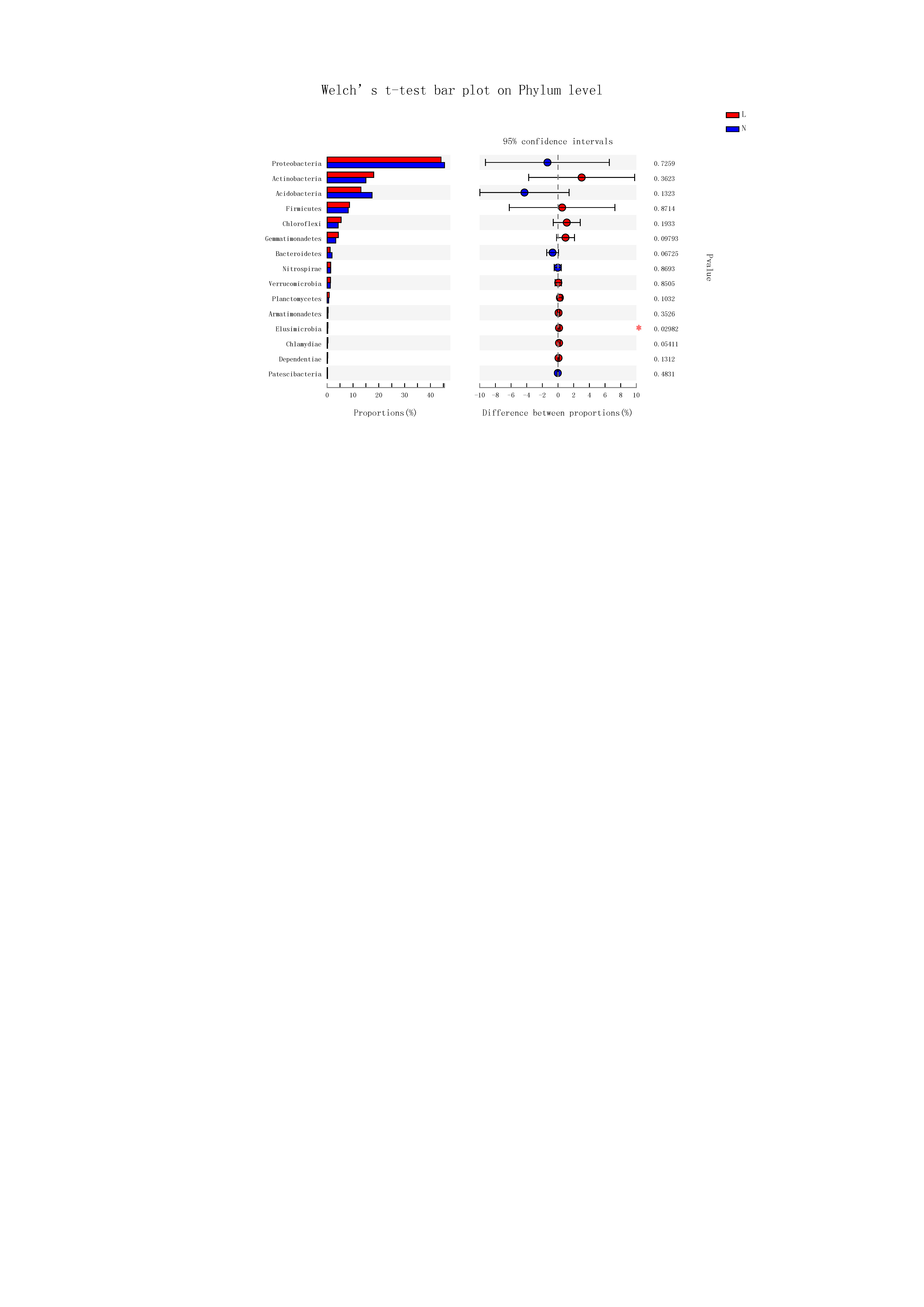

Supplement: Supplementary Figure 1 — The relative abundance of main bacterial community in soils under different treatments. [file Image_1.JPEG]

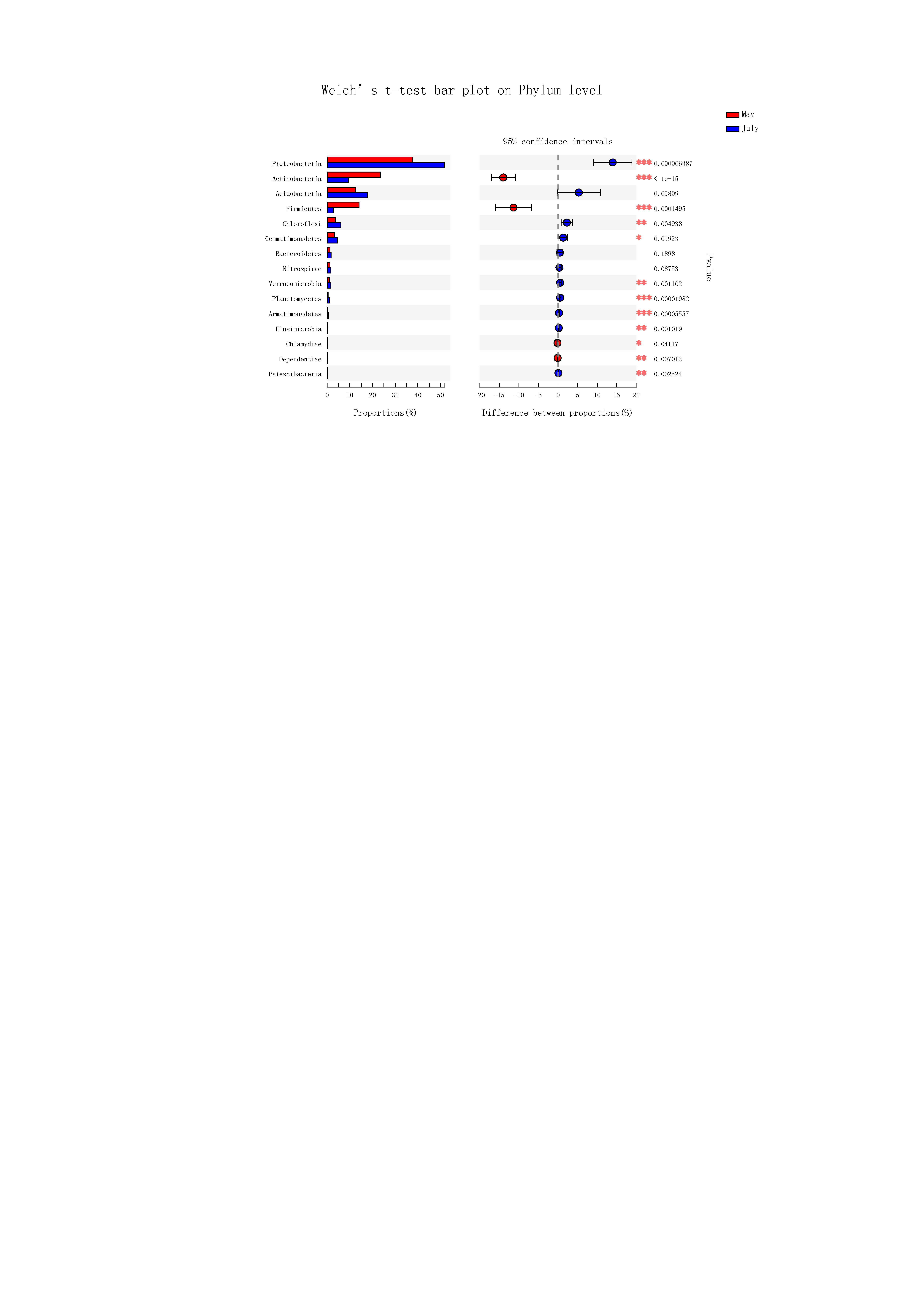

Supplement: Supplementary Figure 2 — The relative abundance of main bacterial community in phylum level under different treatments. [file Image_2.JPEG]

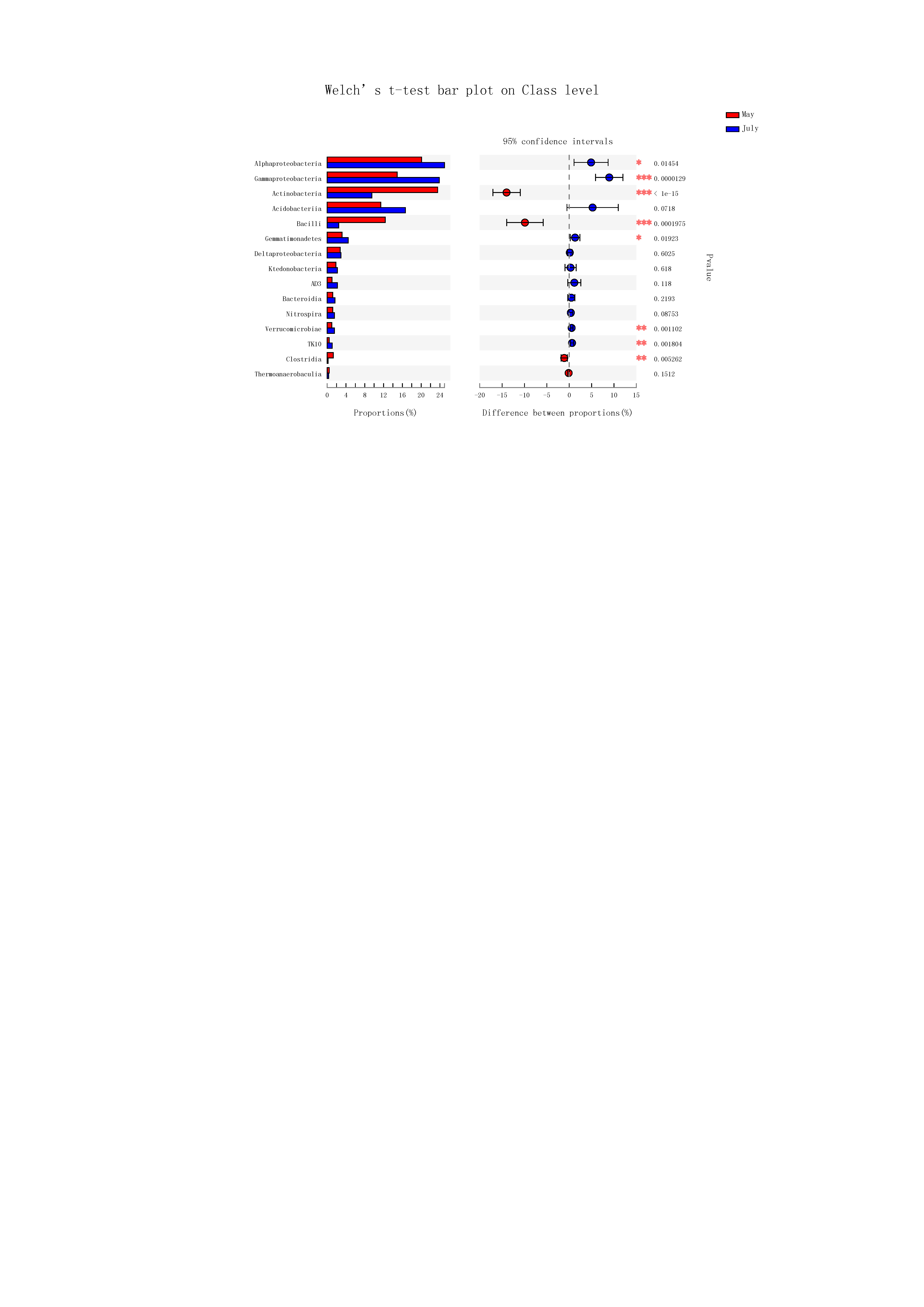

Supplement: Supplementary Figure 3 — The relative abundance of main bacterial community in class level under different treatments. [file Image_3.JPEG]
